# Mucinous Adenocarcinoma of the Prostate With Normal Prostate-Specific Antigen Levels, Pulmonary Metastasis, and the Absence of Nodal Disease: A Case Report

**DOI:** 10.7759/cureus.56563

**Published:** 2024-03-20

**Authors:** Arham A Khokhar, Sarah A Howles, Aaron W Leiblich, Khubaib Samdani, Mubariz Ahmed

**Affiliations:** 1 Urology Department, Churchill Hospital, Oxford University Hospitals NHS Trust, Oxford, GBR; 2 Surgery Department, Benazir Bhutto Hospital, Rawalpindi, PAK; 3 Medicine Department, Isfandyar Bukhari District Hospital, Attock, PAK

**Keywords:** osseous involvement, trans urethral resection of prostate (turp), pirads score, lymph nodes, prostatic specific antigen, multiple pulmonary nodules, mucinous adenocarcinoma, lung metastasis, prostatic cancer, prostate

## Abstract

A 74-year-old man was suffering from nine months of perineal pain and progressive worsening of urinary symptoms including nocturia and urgency. His prostate-specific antigen (PSA) levels were 1.48 ng/mL at the time of referral. Initially, a differential diagnosis of prostatitis or seminal vesicle inflammation was made, and four weeks of antibiotics were prescribed, which were later extended to six weeks due to failure of symptoms to resolve. Magnetic resonance imaging (MRI) of the prostate was then conducted. The impression was that there was ejaculatory duct obstruction caused by enlarged seminal vesicles with no evidence of significant prostate cancer. The prostate-specific antigen density (PSAd) was 0.04, and the prostate imaging reporting and data system (PIRADS) score was I-II.

A CT chest with contrast was conducted for further investigation of pulmonary nodules found on the CT urogram. It revealed multiple calcified pulmonary nodules which were suspicious of malignancy. A CT-guided biopsy of one of the pulmonary nodules was taken, and histopathological analysis revealed a mucinous adenocarcinoma. A transurethral resection of the prostate (TURP) was then performed. Histopathological analysis of the prostatic surgical specimen revealed invasive mucinous adenocarcinoma. Based on the findings, a diagnosis of mucinous adenocarcinoma of the prostate with atypical lung metastasis without osseous or regional lymph node involvement was made, stage T4 N0 M1a. The patient is currently on a treatment regimen consisting of carboplatin, pemetrexed, and pembrolizumab.

## Introduction

Prostate cancer accounts for ~15% of all cancer cases diagnosed in males [1]. It can present as localized or metastatic disease. The most common histopathological subtype is acinar adenocarcinoma, rarer subtypes include ductal adenocarcinoma and mucinous adenocarcinoma [2]. Mucinous adenocarcinoma accounts for only 0.2% to 0.4% of all cancer cases [2].

Atypical presentations of prostate cancer such as mucinous adenocarcinoma, can represent a diagnostic challenge. In this case report we present a case of mucinous adenocarcinoma of the prostate with isolated lung metastases without lymph nodes or osseous involvement, which was diagnosed via histology following a transurethral resection of the prostate (TURP). Only a handful of similar cases have been reported previously [3]. Cancer frequently spreads first to lymph nodes near the original tumor [4], then to organs like the liver, lungs, and bones. Over 40% of men with metastatic prostate cancer develop lung metastases [5]. Still, isolated lung involvement without bone or lymph node spread and a low Gleason score is exceptionally rare, with only a few documented cases [6].

## Case presentation

A 74-year-old male with a history of hypertension and a 25-pack-year history of smoking was referred from primary care via the NHS two-week-wait suspected cancer pathway system to a tertiary care urology department in the United Kingdom. The referral was made for an investigation of new-onset microscopic hematuria. The patient had been previously suffering from nine months of perineal pain and progressive worsening of urinary symptoms that included nocturia and urgency. The patient’s prostate-specific antigen (PSA) level was 1.48 ng/mL at the time of referral.

The patient reported that his pain and urinary symptoms were becoming increasingly debilitating and were impacting his quality of life. He did not report any respiratory symptoms, unexplained weight loss, or loss of appetite. There was no family history of prostate cancer. Digital rectal examination (DRE) revealed a hard mass above the prostate but the prostate itself was reported to be normal.

**Figure 1:**
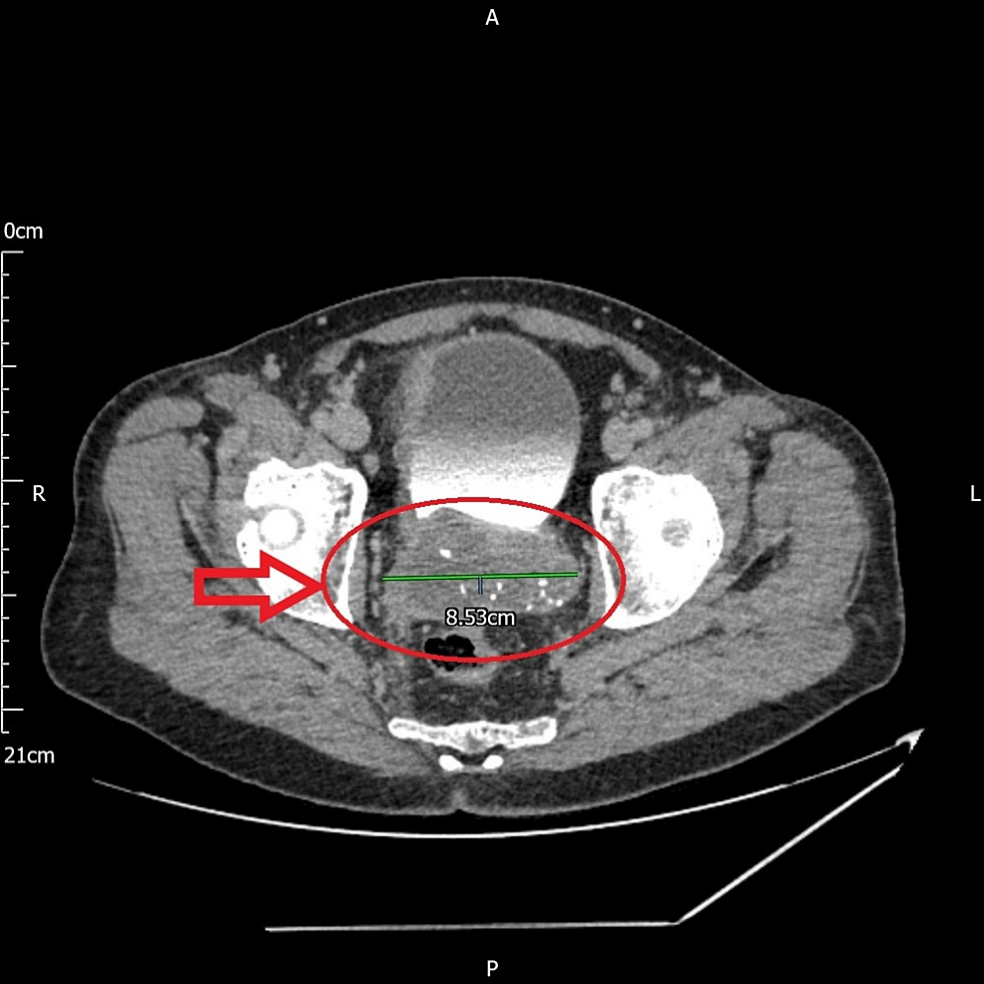
CT urogram showing enlarged seminal vesicles having mixed density with cystic components, heterogeneous enhancement, and coarse calcification

Flexible cystoscopy demonstrated a normal bladder with a moderately occlusive, bilobed prostate. Uroflowmetry showed a Qmax of 10 mL/s, an intermittent/straining pattern, and a voided volume of 368 mL. Post void residual was 248 mL. Computed tomography (CT) urogram showed that the seminal vesicles appeared enlarged, measuring 8.5 cm in maximum transverse diameter, and having mixed density with cystic components, heterogeneous enhancement, and coarse calcification (Figure 1). Incidental findings of bilateral pulmonary nodules were made. Management of the patient's urological issues was commenced by the urology department and a referral was made to the chest medicine clinic for further evaluation of the pulmonary nodules.

**Figure 2:**
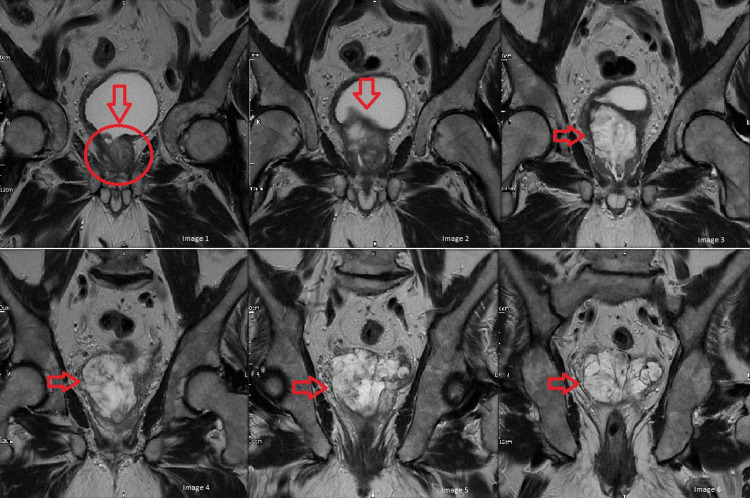
Images 1 to 6 (labeled) are part of a series of MRI images of the prostate showing heterogeneous, calcified mass inseparable from the ejaculatory ducts, distal vas deferens, and seminal vesicles bilaterally. The mass contains a large volume of hemorrhagic/proteinaceous material

In light of the patient’s historical, examination and investigation findings, a differential diagnosis of prostatitis or seminal vesicle inflammation was made by the urology department and four weeks of antibiotics were prescribed. When the symptoms failed to resolve, this prescription was extended to six weeks and Magnetic resonance imaging (MRI) of the prostate was requested. The MRI revealed a heterogeneous, calcified mass inseparable from the ejaculatory ducts, distal vas deferens, and seminal vesicles bilaterally, containing a large volume of hemorrhagic/proteinaceous material (Figure 2). The PSA density (PSAd) was 0.04, and the prostate imaging reporting and data system (PIRADS) score was I-II. The impression was that there was ejaculatory duct obstruction caused by enlarged seminal vesicles with no MRI evidence of significant prostate cancer. A TURP was planned, which would allow for complete pathological analysis and was anticipated to alleviate the patient's symptoms.

**Figure 3:**
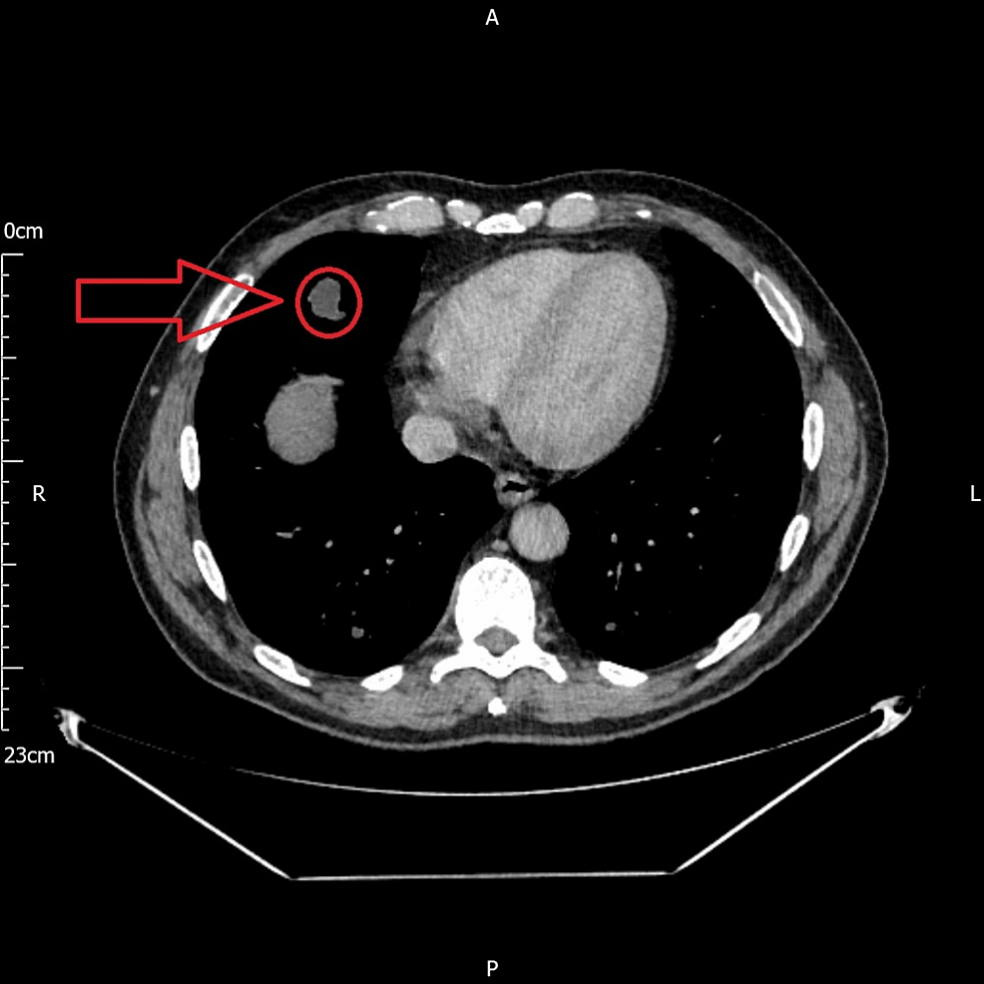
CT scan image showing a right lung middle lobe nodule

**Figure 4:**
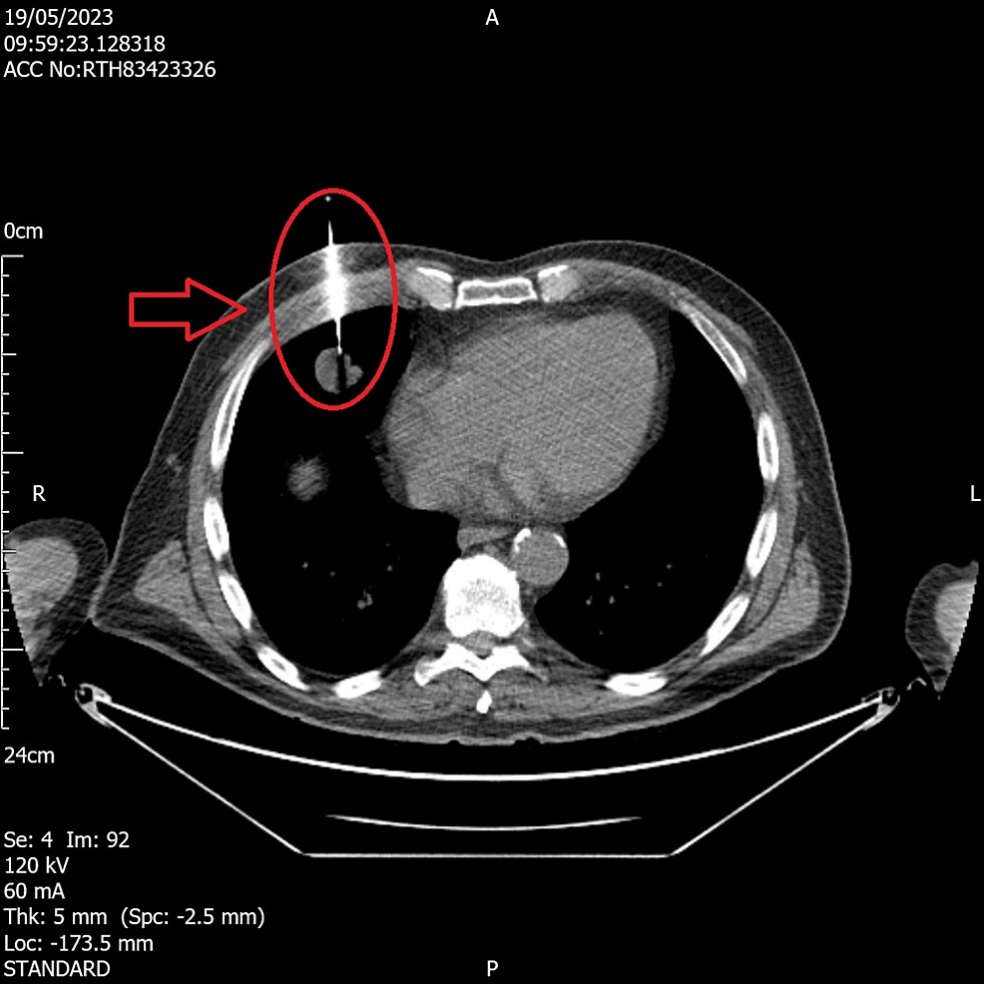
CT-guided biopsy of pulmonary nodule

Meanwhile, the chest medicine clinic found no abnormal pulmonary signs or symptoms. The patient’s lung function tests were normal. CT chest with contrast was requested which revealed multiple calcified pulmonary nodules that were suspicious of malignancy. There was a lobulated 2.2 cm nodule in the right middle lobe with satellite nodularity (Figure 3). There were 2 cm nodules in the posterior aspects of both lower lung lobes, a ground glass nodule measuring 7 mm in the apical right upper lobe, and a 5-mm subpleural nodule in the left upper lobe. There were further multiple tiny subpleural nodules measuring less than 2 mm in diameter, which were indeterminate. The differential diagnosis at this stage was either local metastasis from a primary lung tumor or metastasis from a distant secondary site such as the prostate. A CT-guided biopsy of one of the pulmonary nodules was taken (Figure 4). Histopathological analysis revealed a mucinous adenocarcinoma, with its morphology reminiscent of colorectal adenocarcinoma with elongated nuclei. However, bowel markers including CK20 and CDX2, CK7, and TTF1 were negative; there were insufficient samples for PD-L1, HC (ALK and ROS1), and DNA/RNA panels.

The results of the pulmonary nodule biopsy were discussed in a multi-disciplinary team (MDT) meeting. The MDT included input from the urology, chest medicine, radiology, and histopathology departments. A decision to perform a whole-body fluorodeoxyglucose positron emission tomography-computed tomography (FDG PET-CT) scan was made to look for the extent of malignancy, as well as the potential primary source.

**Figure 5:**
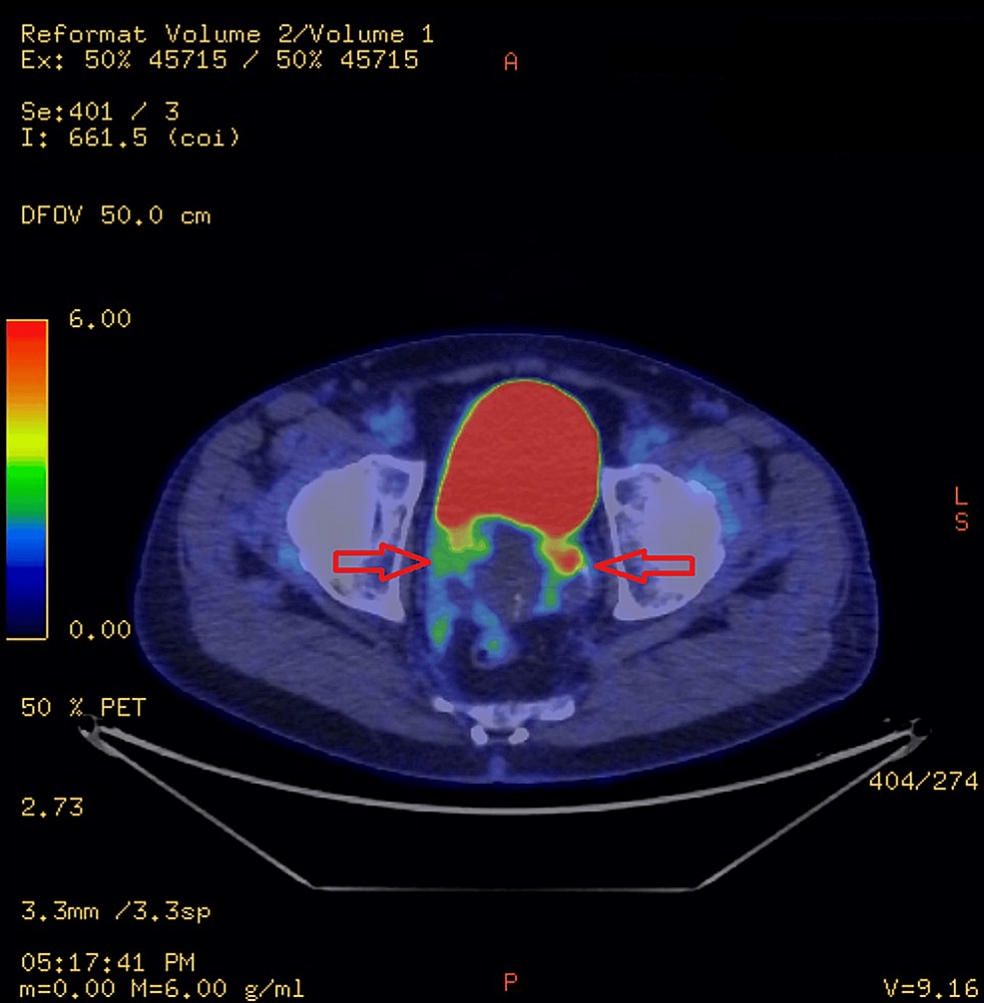
FDG uptake within the left lobe of the prostate and seminal vesicles

**Figure 6:**
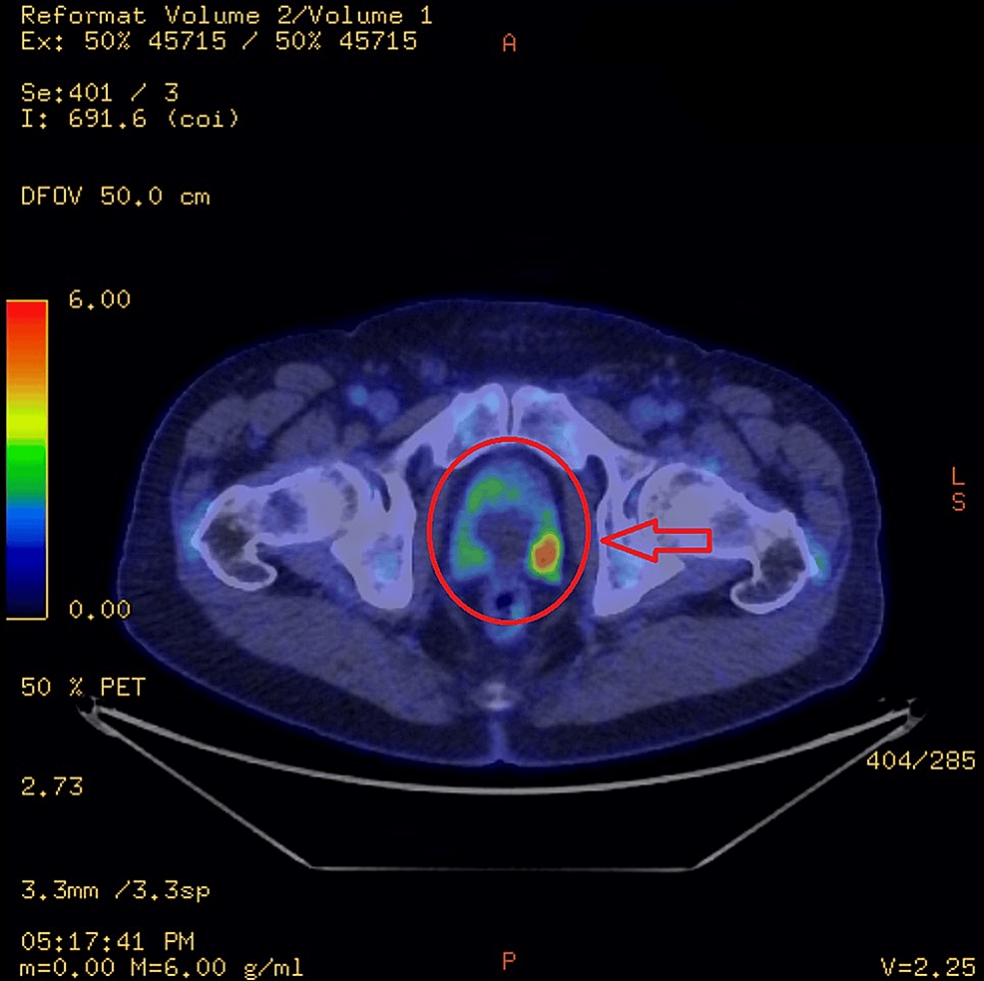
FDG uptake within the left lobe of the prostate and seminal vesicles

**Figure 7:**
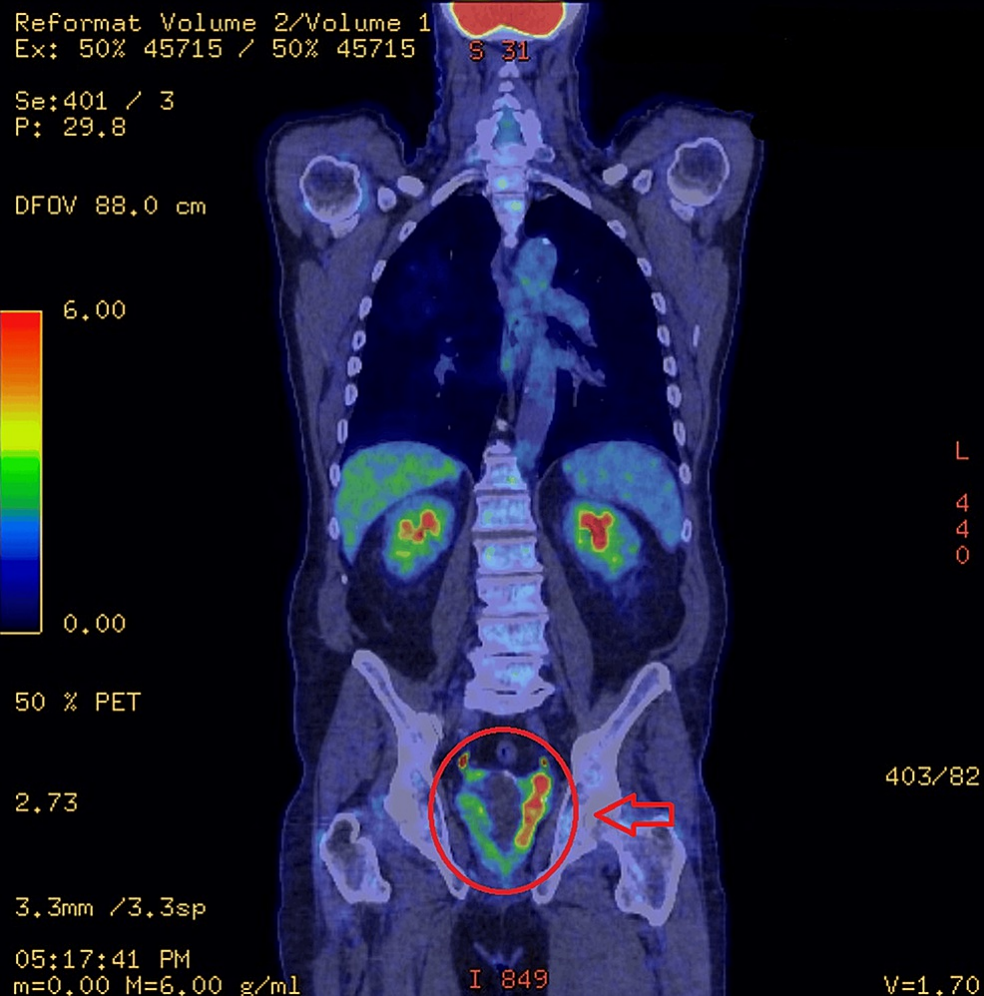
An FDG PET-CT image of the body (notice FDG uptake in prostate and seminal vesicles)

**Figure 8:**
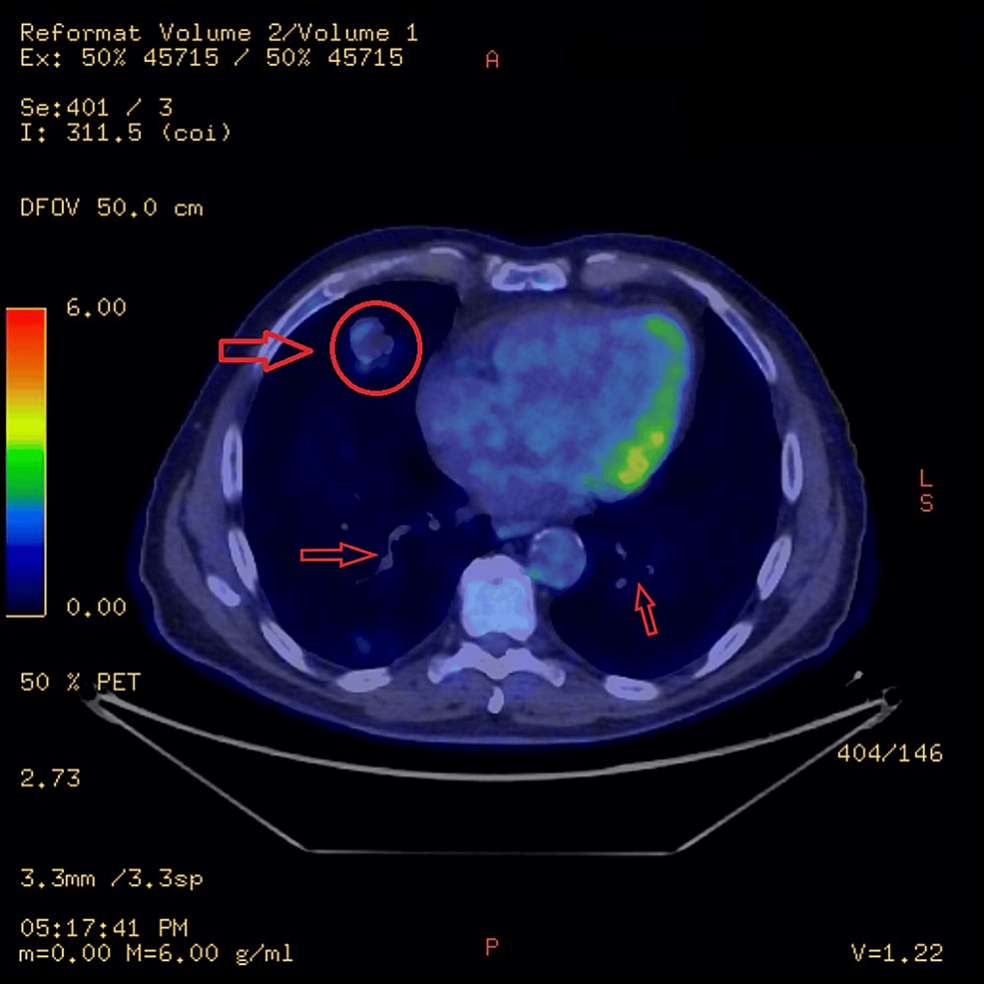
FDG uptake in the lung nodules

The FDG PET-CT scan revealed an enlarged prostate, with the seminal vesicles containing cystic lesions, and enlarged bilateral ureters. There was a moderate FDG uptake within the left lobe of the prostate and seminal vesicles. Findings can be seen in Figures 5-7. There was FDG uptake in the lung nodules as well (Figure 8). The scan revealed no evidence of regional lymph node involvement (there were no FDG avid or enlarged nodes). Moreover, there was no proof of osseous involvement. The potential primary source of malignancy appeared to be the prostate, although the absence of nodal disease was unusual.

The TURP that was previously planned was performed by the urology team, which improved symptoms of nocturia and urgency. Invasive mucinous adenocarcinoma was found on histopathological analysis of the prostatic surgical specimen. Based on the findings described above, the MDT agreed on a diagnosis of mucinous adenocarcinoma of the prostate with atypical lung metastasis without osseous or lymph node involvement, stage T4 N0 M1a. An oncology review initiated treatment with carboplatin, pemetrexed, and pembrolizumab. The patient remains on this regimen currently. 

## Discussion

Mucinous adenocarcinoma of the prostate is a rare condition that can either originate as a primary tumor within the prostate or result from metastasis from other areas such as the bladder, urethra, or colorectal regions [7,8]. In our investigation, it was indicated that the patient likely had a primary prostate tumor. Primary mucinous prostate tumors fall into three categories: mucinous adenocarcinoma of the prostate, prostatic adenocarcinoma with mucinous features, and mucinous adenocarcinoma of the prostatic urethra [4]. Mucinous adenocarcinoma is specifically characterized by the presence of extravasated mucin in more than 25% of the tumor volume in a radical prostatectomy specimen. Consistent with this statement, our pathologist's report revealed the presence of extravasated mucin exceeding 25% of the tumor volume. When the mucinous component occupies less than 25% of the tumor volume, the term “prostatic adenocarcinoma with mucinous features” is appropriate. However, the clinical significance of distinguishing between mucinous adenocarcinoma and adenocarcinoma with less than 25% mucinous involvement remains uncertain [4].

Based on a retrospective study of forty-seven cases of mucinous adenocarcinoma of the prostate identified at radical prostatectomy, the average age at diagnosis of prostatic mucinous adenocarcinoma was 56 years (range, 44 to 69 years) and mean preoperative PSA level was 9.0 ng/mL (range, 1.9 to 34.3 ng/mL) [9]. The patient described here presented at an older age (74 years) and with a lower PSA level (1.48 ng/mL).

Typically, prostatic adenocarcinoma metastasizes to local lymph nodes [10], bones, lungs, and liver [11]. It has been reported that isolated lung metastases, without osseous or lymph node involvement, occur in less than 1% of autopsies involving patients with metastatic prostate cancer [12]. A multinodular pattern of lung metastasis was observed in our patient which may be the result of a hematological spread of the disease [13]. A diffuse interstitial pattern of lung metastasis is more commonly seen in prostate cancer, which is thought to represent lymphatic spread [13]. Hematological spread may also explain the absence of nodal disease in our patient. 

The clinical presentation of prostatic mucinous adenocarcinoma is not markedly different from conventional acinar prostatic carcinoma, and the patients may remain asymptomatic for years despite the presence of the disease [14]. Some studies described that mucinous prostate cancer demonstrates a more aggressive behavior compared to typical acinar adenocarcinoma of the prostate [15,16] while others indicated similar outcomes between these two tumor types [17]. According to research conducted by Zhao et al. using The Surveillance, Epidemiology, and End Results (SEER) database, mucinous prostate cancer exhibits a similar prognosis to typical prostate acinar carcinoma [17]. Nonspecific symptoms, such as obstructive urinary voiding, urinary incontinence, painful micturition, perineal discomfort, nocturia, hematuria, and weight loss may occur [18].

## Conclusions

In summary, we presented a case involving mucinous adenocarcinoma of the prostate with isolated lung metastasis, noteworthy for the absence of osseous or lymph node involvement. The diagnostic process proved challenging due to an apparently normal prostate on DRE, a low PSA level of 1.48 ng/ml, and a PIRADS score of I-II. This highlights the importance for clinicians to maintain awareness of the potential for prostatic malignancy, even when DRE, PSA, and imaging results appear unremarkable. Vigilance is particularly crucial when patients report symptoms such as obstructive urinary voiding, urinary incontinence, painful micturition, perineal discomfort, pollakiuria, nocturia, hematuria, and weight loss.
